# Machine learning in the development of targeting microRNAs in human disease

**DOI:** 10.3389/fgene.2022.1088189

**Published:** 2023-01-04

**Authors:** Yuxun Luo, Li Peng, Wenyu Shan, Mengyue Sun, Lingyun Luo, Wei Liang

**Affiliations:** ^1^ School of Computer Science and Engineering, Hunan University of Science and Technology, Xiangtan, China; ^2^ Hunan Key Laboratory for Service computing and Novel Software Technology, Xiangtan, China; ^3^ School of Computer Science, University of South China, Hengyang, China; ^4^ School of Polymer Science and Polymer Engineering, The University of Akron, Akron, OH, United States

**Keywords:** machine learning, mirna therapy, miRNA-disease association, miRNA-drug association, deep learning

## Abstract

A microRNA is a small, single-stranded, non-coding ribonucleic acid that plays a crucial role in RNA silencing and can regulate gene expression. With the in-depth study of miRNA in development and disease, miRNA has become an attractive target for novel therapeutic strategies. Exploring miRNA targeting therapy only through experiments is expensive and laborious, so it is essential to develop novel and efficient computational methods to narrow down the search. Recent advances in machine learning applied in biomedical informatics provide opportunities to explore miRNA-targeting drugs, thus promoting miRNA therapeutics. This review provides an overview of recent advancements in miRNA targeting therapeutic using machine learning. First, we mainly describe the basics of predicting miRNA targeting drugs, including pharmacogenomic data resources and data preprocessing. Then we present primary machine learning algorithms and elaborate their application in discovering relationships among miRNAs, drugs, and diseases. Along with the progress of miRNA targeting therapeutics, we finally analyze and discuss the current challenges and opportunities that machine learning confronts.

## 1 Introduction

As a kind of non-coding RNA transcript, MicroRNA (miRNA) plays a vital role in cell proliferation, survival and differentiation by modulating the transcription of target messenger RNA (mRNA) and disrupting the translation of mRNA (Rupaimoole and Slack, 2017). The miRNA-mRNA interactions usually lead to translation inhibition or mRNA degradation, which brings about the reduction of the final protein output (Guo et al., 2010). MiRNAs act as novel therapeutic targets and potential diagnostic markers due to they can regulate gene expression involved in the pathogenesis of cancer and other complex diseases (Tay et al., 2008). Just a few years after the first miRNA was discovered by Lee and others in 1993 (Lee et al., 1993), the research of miRNA biology dramatically bloomed. The experimentally validated function of miRNAs laid a solid foundation for cellular biology, which enables researchers to study associated diseases and drugs at the molecular level (Chen et al., 2020).

The efficacy of various miRNA therapies depends on the accurate relationships between miRNAs and diseases. There are many validated relationships that exist between miRNAs and prevalent diseases, such as lung cancer, pancreatic, ovarian cancer, and so on (Roldo et al., 2006). For example, excisions and downregulation of the miR-15/16 cluster frequently occur in chronic lymphocytic leukemia (Calin et al., 2008), and the significant upregulation of miR-21 is involved in hematological malignancies (Fulci et al., 2007). The decreased expression of human let-7 miRNA family in lung cancer was associated with poor prognosis in patients (Takamizawa et al., 2004). MiRNAs also have been associated with several metabolic pathways (Fernández-Hernando et al., 2013), for example, miR-33 influenced the level of triglyceride and the high-density lipoprotein in serum (Marquart et al., 2010). However, it costs a lot of time, money, and resources to acquire associations verified in experiments, which brought about a widespread interest in the computational discovery of underlying miRNA-disease associations during the last few years. More than 186,000 related articles were available online, and many relevant databases and models were designed (Huang et al., 2022a). For instance, the latest version of Human MicroRNA Disease Database (Huang et al., 2019) records 35,547 entries, and the commonly used database miR2Disease (Jiang et al., 2009) contains 3,273 associations. Meanwhile, based on the conception that similar miRNAs would be associated with similar diseases, various computational models were adopted to identify underlying associations. Usually, the homogeneous network and the heterogeneous network were built to extract desired feature embeddings *via* machine learning methods (Fu and Peng, 2017).

The therapeutic advance of diseases was deeply influenced by the time-consuming and costly process of drug discovery and development. Most drugs generally are small molecules, namely low molecular weight organic compounds, that act as a regulator in a biological process. It was indicated in studies that small molecules could disrupt protein interactions, and also suppress specific functions of a multifunctional protein; hence it may have a positive effect on diseases (Melo et al., 2011). Unlike biologics with which injection and other parenteral administration are usually required, most small-molecule drugs can be taken orally. The urgent request for novel therapeutic alternatives makes the approach of targeting disease-related miRNA with small molecules seem to be promising. Since Gumireddy et al. (2008) developed the first small molecule inhibitor of miRNA for specifically suppressing miR-21, numerous miRNA inhibitors have been discovered *via* a sequence-based computational approach or high throughput screening (Young et al., 2010). For instance, the miR-122 inhibitors were identified to suppress the miR-122 expression and reduce 50% of HCV viral load *in vitro* (Kutay et al., 2006). Besides, streptomycin, neomycin, tobramycin, and amikacin could impede miR-27a function, which plays a role in the regulation of adipogenesis, gastric cancer and so on, by directly interacting with pre-miR-27a (Zhang et al., 2011; Chandrasekhar et al., 2012). Recently, more and more miRNA-drug association research has been launched, such as the Developmental Therapeutics Program funded by the National Cancer Institute of United States, which publicly published related datasets. Similarly, many computational methods based on regression, matrix factorization, neural networks and so on have been proposed.

In this review, we firstly listed several manually curated mainstream databases of miRNA-disease associations and miRNA-drug associations as comprehensive resources for computational approaches. Then, with the rapid bloom of machine learning approaches, we reviewed some representative studies on predicting underlying relationships between miRNAs and diseases or drugs using modified learning models. Due to the length limit of the paper, not all papers related to the above introduction are able to be included. Nevertheless, we collected the commonly used databases and the most representative computational methods to reveal promising development trends for targeting miRNAs in human diseases and drugs.

## 2 Database

As we all know, miRNA expression deregulation is crucial to the state transition from a physiological to a pathological one. Many studies in recent have suggested that bioactive drugs can act as the regulator of miRNA expression, hence indicating a new therapy that miRNAs targeted with small molecules. Therefore, more and more diversified databases containing various omics data increased dramatically due to the development of system biology and molecular biology. The database of miRNAs-diseases was generated from experimentally validated miRNA-disease associations, and the miRNAs-drugs databases originated from experimentally verified small molecules’ impacts on the expression of microRNA. In this section, we concluded data details in the most popularly used and commonly cited databases, most of which were still in maintained status, from aspects of miRNA-diseases and miRNA-drugs. Table 1 listed various information about these mainstream databases.

**TABLE 1:** Main databases for accelerating miRNA therapy based on machine learning.

Database	Published year (latest update)	Data type	Number of data	URL
miR2Disease	2008 (2022)	Relationships between deregulated miRNAs and diverse human diseases	3273 entries, 349 microRNAs, 163 human diseases	http://www.mir2disease.org/
PhenomiR	2009 (2011)	Differential regulation of miRNA expression in diseases	11029 data points and 572 miRNAs	http://mips.helmholtz-muenchen.de/phenomir
miRGen	2007 (2020)	miRNAs related to disease status information	Over 1500 miRNAs and 133 cell lines, primary cells, and tissues	http://www.microrna.gr/mirgenv4
miRmine	2016 (2016)	miRNA expression profiles in tissues, cell lines, and diseases	304 miRNA sequencing datasets for 15 tissues and 24 cell lines	https://guanfiles.dcmb.med.umich.edu/mirmine
miRTarBase	2011 (2022)	miRNA-associated diseases and the relationship between miRNA-target interactions and disease	4630 miRNAs and 30tissues/cell lines from 440CLIP-seq datasets	https://miRTarBase.cuhk.edu.cn/
HMDD	2007 (2022)	miRNA-disease associations could be divided into 6 categories of genetics, target, circulation, tissue, epigenetics, and others	35547 entries of miRNA-disease association between 1206 miRNA genes and 893 diseases	http://www.cuilab.cn/hmdd
Pharmaco-miR Verified Sets	2013 (2013)	miRNA pharmacogenomic sets that were verified in experiments	119 target genes, 72 drugs (whose function depends on the gene), and 105 miRNAs	www.Pharmaco-miR.org
SM2miR	2012 (2015)	The experimentally verified small molecules’ effects on miRNA expression	4989 entries of relationships between 1658 miRNAs and 255 small molecules	http://www.jianglab.cn/SM2miR/
DTP NCI-60 dataset	2016 (2022)	A dataset of CellMiner database which screened over chemical compounds by utilizing diverse human cancer cell lines	335 miRNA expressions and half-cell growth inhibition concentration from 18724 drugs	https://discover.nci.nih.gov/cellminer
ncDR	2017 (2017)	miRNA-drug resistance associations for predicting non-coding RNA related to drug resistance	5864 experimentally verified relationships between 145 drug compounds and 877 miRNAs	http://www.jianglab.cn/ncDR

The 1st column gives the database names. The 2nd column presents the published year and the latest update of the database. The 3rd column introduces data type included in the database. The 4th column presents the number of data. The 5th column introduces the URL of the database.

### 2.1 miRNA-disease associations

#### 2.1.1 miR2Disease

To date, the latest version of miR2Disease (Jiang et al., 2009) curated 3,273 relationships between 349 human microRNAs and 163 human diseases, one-eighth of which suggested the pathogenic roles of various human diseases related to miRNA deregulation. Resources in the miR2Disease contained various details about microRNA-disease relationships, in which every entry could be retrieved by disease name, miRNA ID, or target gene. Additionally, the literature reference, the detection method for miRNA expression, the expression pattern of miRNA, and a brief description of a relationship are also included in this database.

#### 2.1.2 PhenomiR

The PhenomiR database (Ruepp et al., 2010) included 11,029 data points and 572 miRNAs, which were collected from 542 related studies focusing on the differential regulation of miRNA expression in diseases. In addition to some usual information, PhenomiR provided in-depth information such as the sample size, the quantitative fold-change of miRNA expression, and the origin analysis of samples (cell culture or patients). Depending on disease type in the PhenomiR dataset, we can contrast conclusions originating from patient studies with independent resources drawn from cell culture studies.

#### 2.1.3 miRGen

The latest version miRGen v4 (Perdikopanis et al., 2021) uniquely integrated annotations for numerous cell-specific miRNA promoters with transcription factor binding sites derived from experiments, which clearly revealed the regulation of miRNA at the transcriptional level. Combined with more than 1,000 cap analyses results from gene expression samples (Shiraki et al., 2003) of 133 cell lines, primary cells, and tissues derived from the FANTOM Consortium (Forrest et al., 2014), miRNA transcription start sites that specific in cell type were provided for more than 1500 miRNAs. Details in this database can be queried through the sample-oriented method or miRNA-oriented method.

#### 2.1.4 miRmine

The miRmine database (Panwar et al., 2017) contained details of different miRNAs and collected expression profiles from various miRNA databases. The miRmine functionality included searches based on miRNA and cell-line/tissue, comparison of multiple miRNAs, normal and human disease information, and so on. For specific tissue or cell-line type, miRmine could retrieve single or multiple miRNAs expression information. Besides, retrieved results could be shown in various graphs and interactive formats.

#### 2.1.5 miRTarBase

The miRTarBase 9.0 (Huang et al., 2022b) released in 2021 documented over 360,000 miRNA-target interactions between 27,172 targets and 4,630 miRNAs collected from 13,389 related studies, which facilitated the research of miRNAs’ function in pathology and promoted the improvement of diagnostic and therapeutic tools. Integrating with increasing miRNA expression and biological data, miRTarBase accumulated miRNA-target interactions verified in experiments and satisfied biologists’ requirements. Additionally, an optimized scoring system is utilized in the updated version to reinforce the important identification of related articles and relevant disease information.

#### 2.1.6 HMDD

To date, 35,547 entries of miRNA-disease association between 1,206 miRNA genes and 893 diseases curated from 19,280 papers were collected in HMDD (Huang et al., 2019). Disease network analysis modules were applied in the latest HMDD v3.3, which was released in Sep 2022. Covering 20 kinds of detailed evidence code derived from literature, miRNA-disease associations in HMDD were divided into six categories of genetics, target, circulation, tissue, epigenetics, and others. Due to the wide coverage and abundant experimentally verified associations, HMDD became one of the most popular databases regarding association prediction and was widely adopted as the benchmark in training and testing prediction models.

### 2.2 miRNA-drug associations

#### 2.2.1 Pharmaco-miR Verified Sets

In 2014, Pharmaco-miR Verified Set (Rukov et al., 2014) manually curated 269 miRNA pharmacogenomic data from 149 original literature. It is a dataset of miRNA pharmacogenomic sets that were verified in experiments, containing119 target genes, 72 drugs (whose function depends on the gene), and 105 miRNAs. In Pharmaco-miR Verified Sets, the miRNA directly targeted the gene in a specified context, which was typically exhibited *via* luciferase experiments. Meanwhile, in the same context, the efficacy of drugs was affected by the subsequent suppression of gene expression in this database.

#### 2.2.2 SM2miR

SM2miR (Liu et al., 2013) collected miRNA expression influenced by experimentally verified small molecules’ effects in 21 species curated from the published papers. To date, it documented 4,989 entries of relationships between 1,658 miRNAs and 255 small molecules. Various details of each entry encompass species, the miRNA expression pattern, accession number in miRbase and DrugBank, detection conditions, experimental method, PubChem Compound Identifier, PubMed ID, and the related reference information.

#### 2.2.3 DTP NCI-60 dataset

The U.S. National Cancer Institute launched the Developmental Therapeutics Program, which screened over 100,000 chemical compounds by utilizing 60 diverse human cancer cell lines, namely DTP NCI-60 (Blower et al., 2007). In NCI-60 dataset, data consists of 335 miRNA expressions and half-cell growth inhibition concentration (GI50) from 18,724 drugs. The DTP NCI-60 dataset can evaluate the correlations between miRNA expression and drug sensitivity by calculating the Pearson correlation coefficient between miRNA expression level and GI50 value.

#### 2.2.4 ncDR

In 2017, a comprehensive database called ncDR documenting miRNA-drug resistance associations was released to predict non-coding RNA related to drug resistance (Dai et al., 2017). This database contains 5,864 experimentally verified relationships between 145 drug compounds and 877 miRNAs through manually curating from about 3,300 relevant literatures. In addition, 226,109 predicted relationships between drug resistance and miRNA were already provided in this database.

## 3 Predicting miRNA-disease associations

**FIGURE 1:**
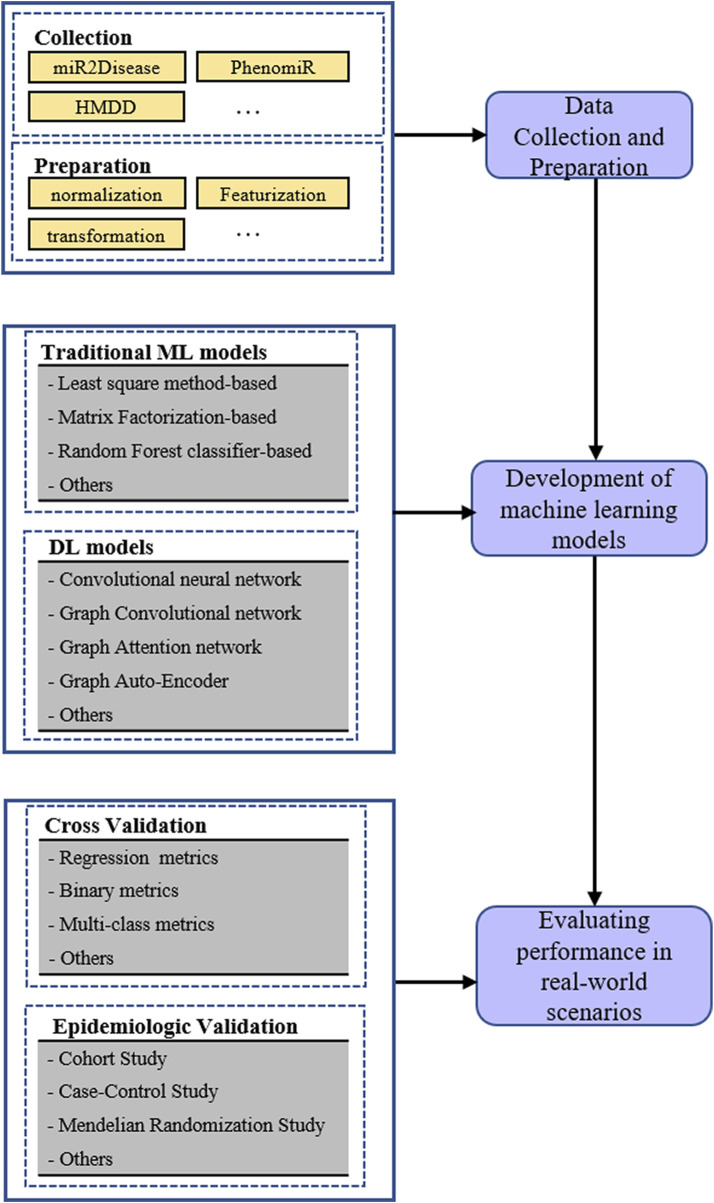
The processes of machine learning models for predicting miRNA-disease associations.

In past biological experiments, plenty of relationships between diseases and miRNAs have been verified, which laid the foundation for discovering latent miRNA-disease associations *in silico*. At first, both negative and positive samples were included in the training set because the association prediction was usually processed as a binary classification task. Undoubtedly, the known miRNA-disease associations constituted positive training samples; hence, negative ones were randomly sampled from the remaining. The remaining set may contain unknown disease miRNA. As we all know, negative samples should only contain miRNAs and diseases between which the relationship was actually nonexistent; however, there are still many unknown miRNA-disease associations that have not been detected in biological experiments. It is most likely that the current negative samples contained many undiscovered associations. Therefore, to avoid bias brought by the sample, various computational methods only learned from verified associations were proposed to accurately predict miRNA-disease associations. Furthermore, the miRNA-disease association prediction was processed as a triplet classification in machine learning approaches, which could identify the role miRNA played. The main process for predicting miRNA disease associations based on machine learning is presented in Figure 1.

### 3.1 Traditional machine learning models for miRNA disease associations

As an example of using a negative training sample, a previous study (Ji et al., 2020) learned graph representations with global structure knowledge in a heterogeneous network consisting of the known associations among miRNA, disease, drug, and protein. Integrating these embeddings with miRNA sequences, disease semantic similarities and so on, a classifier based on Random Forest was applied to discover underlying relationships between miRNAs and diseases.

Meanwhile, more and more approaches preferred to predict unknown miRNA-disease associations only with known ones, so researchers utilized verified associations, such as miRNA-disease, miRNA-gene, and weighted gene-gene, to construct a regularized framework for inferring the latent miRNA-disease associations (Peng et al., 2017a). Similarly, using the identified disease-associated miRNA information, (Luo et al., 2018) built a semi-supervised classifier to calculate the probability of a miRNA related to a given disease, and also utilized graph regularization to avoid overfitting. Considering the sparsity of known data, have also (Luo et al., 2016) proposed a transductive learning-based collective prediction method in which the relevance score was calculated and updated *via* the disease-miRNA network.

To adequately discover disease-related candidate miRNAs, in (Ding et al., 2018) for example, a heterogeneous disease-gene- miRNA network consisting of three types of nodes and five types of links was built to predict associations *via* a regression-based model. For fully utilizing verified miRNA-disease associations. In (Pan et al., 2019), the miRNA-disease associations were synchronously predicted and updated *via* a multi-label, graph-based model, which firstly introduced a set of kernel matrices and then adaptively obtained two optimal kernel matrices. Considering the inherent noise in current databases, a study in (Liang et al., 2019) adaptively learned an affinity graph from various similarity profiles and simultaneously updated the prediction *via* multi-label learning. According to the latest version of HMDD, a study in (Liang et al., 2018) obtained the semantic similarities of disease and function similarities of miRNA. Then, the similarity matrices and association matrix were iteratively updated to generate the optimized association outcome.

Matrix factorization, a method of multiplying two different entities to generate potential features, is another essential method for predicting miRNA disease associations. As in (Peng et al., 2017b) for example, a matrix recovery approach was utilized to integrate the weight matrix to recover association matrix; hence novel latent associations were accurately inferred without the need for negative samples. Integrated with the label propagation algorithm, a study in (Peng et al., 2022) adopted robust nonnegative matrix factorization to predict underlying associations more precisely. To be specific, using the integrated similarity information, the original adjacency matrix was updated *via* matrix multiplication to reduce the influence of negative samples. For sparse existing associations and new diseases or miRNAs, a previous work (Xiao et al., 2018) developed a preprocessing step that built the interaction score profiles to facilitate prediction, and then utilized graph regularized non-negative matrix factorization based on integrated multisource data to discover underlying associations.

Although most methods *in silico* currently focus on discovering unknown miRNA-disease associations, there are some approaches that could identify the multiple relationship types among various associations as the roles miRNAs played in diseases significantly diverged. For example, the down-regulation of mir-16 and mir-15 could induce chronic lymphocytic leukemia in B cell (Calin et al., 2002), while the different expression of serum miRNAs, such as mir-1307-3p, mir-1246 and so on, could assist researchers in tracing breast cancer early (Shimomura et al., 2016). To this end, a more recent study (Huang et al., 2021) innovatively constructed a tensor composed of miRNA-disease-type triples, and then adopted tensor decomposition that utilized the similarity information as decomposition constraints to detect multi-type of miRNA-disease associations. Another study built a novel model for miRNA-disease-type associations by applying tensor robust principal component analysis (Yu et al., 2021a).

### 3.2 Deep learning models for miRNA disease associations

Currently, many prediction methods extracted feature embeddings as the input of convolutional neural networks (CNN). Xuan et al. (2018) constructed a dual convolutional neural network, which was divided into the left and right part, to detect underlying associations. The left CNN learned the integrated feature embedding of original information to produce an association score, and the right learned the feature embedding of the network topology to generate the other score. On this basis, a work in (Xuan et al., 2019) firstly projected nodes of miRNAs and diseases into a low dimensional space to obtain feature embeddings, and then utilized network representation learning and two CNN to discover latent disease-associated miRNAs. In (Peng et al., 2019), the low dimensional feature embeddings were selected by an auto-encoder from a three-layer network consisting of multisource data. Then, the association score was calculated by a deep CNN structure, including the fully-connected layer, max-pooling layer, and convolutional layer.

Besides, some Graph Convolutional Network (GCN) based end-to-end models were also implemented to capture candidate associations. In 2020, a work (Li et al., 2020) respectively learned underlying feature embeddings derived from the miRNA function similarity network and the disease semantic similarity network with GCN encoders. Then an association matrix completion was generated from a novel neural inductive model that adopted learned embeddings as input. As in (Chu et al., 2021), a miRNA-disease pair was regarded as a node in homogeneous graphs, which were easier to learn. Then based on graph sampling, the modified GCN algorithm was implemented on the topology and feature graph to cluster similar nodes. Meanwhile, some other graph neural network methods were also employed in this regard. A graph attention network-based method (Li et al., 2022) aggregated different neighbor information with varying weights to obtain the non-linear features of miRNAs and diseases. Combined with the linear features constructed by correlation profiles, latent miRNA-disease associations were inferred *via* the random forest algorithm. In 2021, Li et al. (2021a) developed an end-to-end framework based on a novel graph auto-encoder model to discover unknown associations. This model aggregated nodes’ neighborhood information *via* a graph neural network-based encoder, which consisted of the multi-layer perceptron and aggregator function, to obtain low dimensional embeddings and effectively integrate heterogeneous information.

Some methods aimed at predicting type instead of taking association prediction as a binary task. In (Huang et al., 2021) for example, miRNA-type- disease triples were innovatively regarded as a tensor, and then tensor decomposition with relation constraints was implemented to complete the type prediction task. Similarly, a more recent work (Yu et al., 2022) could identify dysregulation, downregulation, or upregulation relationship between miRNA and disease because a depth graph representation learning model was trained based on a knowledge graph constructed by extracting disease-miRNA-type triples from existing databases and numerous experimental data.

To fully understand the synergistic effect of miRNA-miRNA pairs on the pathogenesis of complicated diseases, a study (Luo et al., 2021) proposed a new tensor decomposition model based on a graph attention network to discover potential miRNA-miRNA pairs related to diseases. The graph attention network aggregated the feature embeddings from the miRNA function similarity graph, disease semantic similarity graph, and miRNA sequence similarity graph. With the aggregated feature embeddings, the deep tensor factorization was implemented to reconstruct the association tensor consisting of miRNA-miRNA-disease triples.

## 4 Predicting miRNA-drug associations

**FIGURE 2:**
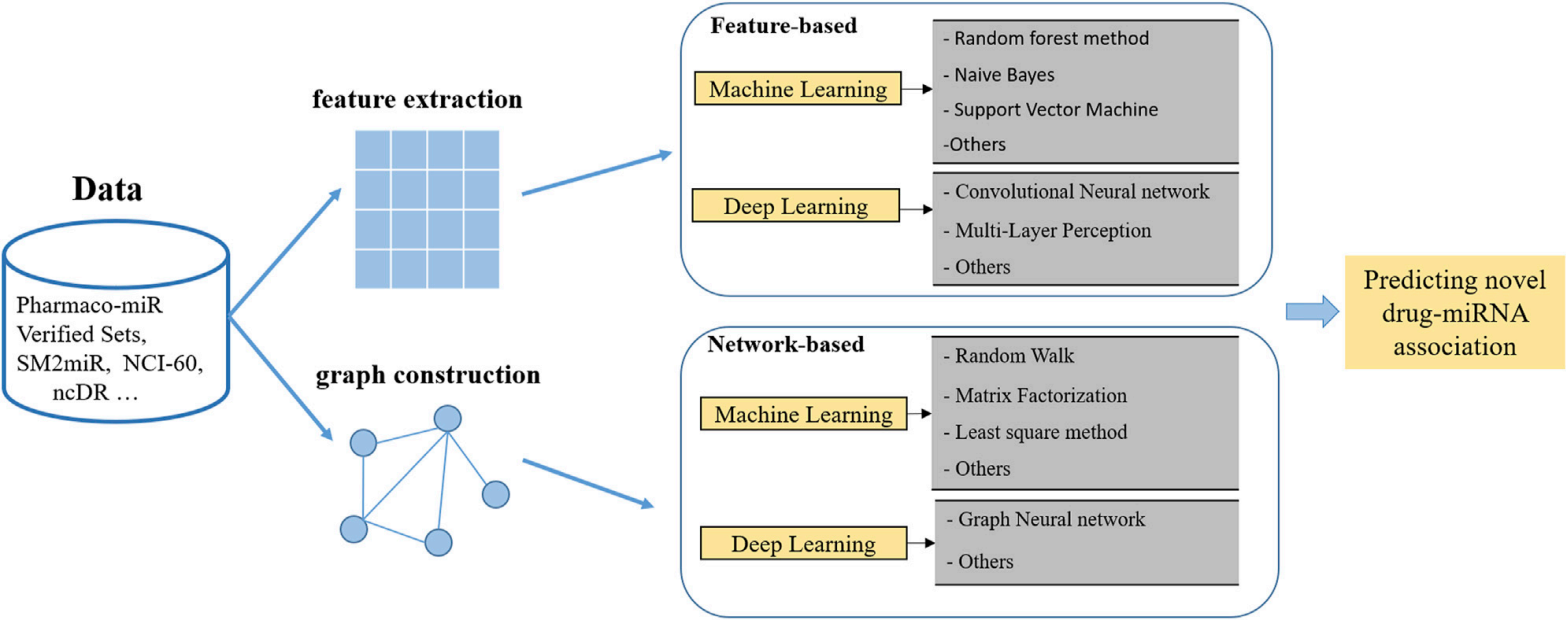
The process of computational models for identifying miRNA-drug associations.

With the accumulated research on miRNA-small-molecule interactions, computational approaches attract more and more attention because they can efficiently promote miRNA-targeted drug discovery and optimization when compared to conventional routine. Varieties of computational models were proposed to discover latent miRNA-drug candidates. Generally speaking, they can be classified into two kinds of approaches for predicting: the traditional machine learning method and the deep learning method, as shown in Figure 2.

### 4.1 Traditional machine learning models for miRNA drug associations

Some machine learning methods focused on constructing novel feature engineering with varied features. A random forest prediction model (Wang et al., 2019) adopted similarities of miRNAs and small molecules as features to accurately predict associations. Specifically for cancer, (Li et al., 2021b) innovatively concatenated features extracted from small molecule structures, miRNA sequences, and cancer symptoms to obtain a new feature vector. Then a random forest model was utilized to predict latent cancer-miRNA-small molecule associations. Similarly, Jamal et al. (2012) developed a prediction model by utilizing Naïve Bayes and Random Forest. In 2017, a work (Xie et al., 2017) was proposed to discover the influential miRNA on the drug *via* the support vector machine, in which feature vectors were drug-miRNA pairs extracted from the related literature.

There are some methods based on random walk algorithm to identify latent miRNA-small molecule associations. In (Liu et al., 2020) for example, Random Walk was utilized in a triple-layer heterogeneous network of disease-miRNA-small molecule association after computing similarities and selecting negative samples. Similarly, a restart algorithm-based Random Walk (Lv et al., 2015) was implemented in a comprehensive network, in which miRNA-miRNA associations, small molecule interactions, and verified miRNA-small molecule targeting pairs were integrated. Meanwhile, some other methods are based on regression algorithm. In Chen et al. (2021) for example, a matrix was defined to represent a heterogeneous network consisting of small molecule similarity, miRNA similarity, and verified miRNA-small molecule associations. Then, the model of the Alternating Direction Method of Multipliers was designed to minimize the nuclear norm of the matrix and obtain predicted scores of underlying miRNA-small molecule associations. Likewise, a work (Wang et al., 2022) developed a prediction model based on the Ensemble of Kernel Ridge Regression. They integrated feature dimensionality reduction with ensemble learning to discover latent small molecule-microRNA associations.

It can be seen in various studies that many computational models adopted matrix factorization. In Yin et al. (2019) for example, a sparse learning method (SLM) was proposed to eliminate noises and improve performance. After the small molecule-miRNA adjacency matrix was decomposed by SLM, latent miRNA-small molecule associations would be obtained *via* a heterogeneous graph that integrated the similarities of miRNAs and small molecules with the improved association information. At the same time, Wang and Chen (2019) not only adopted similarities of small molecules, miRNAs, and diseases but also integrated with associations between miRNAs and diseases/small molecule. Therefore, a three-layer network was built to obtain potential representations of small molecule-miRNA association *via* in-layer similarities and cross-layer associations. Then cross-layer dependency inference on the three-layer network was utilized to identify unknown miRNA-small molecule associations. In addition, the model adopted a regularized optimization to avoid overfitting. Afterward, a study (Zhao et al., 2020) applied matrix decomposition in integrated similarity matrixes and obtained small molecule-miRNA pair similarity by calculating the Kronecker product. Additionally, regularized least square method was applied to acquire the mapping relationships between associated probabilities and miRNA-small molecule pairs. Considering the functional similarity of two miRNAs, clinical similarity and chemical similarity of small molecules, a work (Luo et al., 2020) adopted a nonnegative matrix decomposition method for discovering the potential miRNA-small molecule associations. Besides, combining small molecule-disease associations with miRNA-disease associations, Shen et al. (2020a) adopted graph regularization techniques and the iterative approach in a heterogeneous network to obtain the prediction scores of miRNA-small molecule pairs. In Shen et al. (2020b), the prediction performance was improved by a Restricted Boltzmann Machine-based joint learning framework, which integrated miRNA sequence, heterogeneous network knowledge, and small molecule structure data.

### 4.2 Deep learning models for miRNA drug associations

Currently, Graph Convolution Network is commonly used to process node classification tasks in the homogeneous network. In Huang et al. (2020) for example, a three-layer latent factor model based on graph convolution was developed to discover unknown miRNA-drug resistance associations. In this end-to-end learning scheme, they could not only utilize high-dimensional attributes but also learn graph embedding features of miRNAs/drugs. To overcome the problem of over-smoothing in conventional graph convolution networks, a work (Yu et al., 2021b) simplified GCN by constructing the embedding propagation layer utilizing a weighted sum aggregator. Then, the ideal representations were obtained by summing over the embeddings in each layer. At last, they applied the inner product to discover the unknown miRNA-drug sensitivity associations. Wang et al. (2021) firstly extracted drug/miRNA representations *via* a layer attention graph convolution network in the heterogeneous network consisting of known drug similarities, miRNA similarities, and drug-miRNA interactions. Then they obtained the drug/miRNA embedding vectors by concatenating their representations with drug features derived from drug molecular graphs, and the miRNA expression features, respectively. In addition, they utilized compressed tensor network, tensor decomposition, and multi-layer perceptron to extract node-pair embeddings. Eventually, the potential relationship between miRNA and drug resistance was predicted by the completely connected layer with concatenated representations. Similarly focused on prediction for the relationship of miRNA-drug resistance (Zhao et al., 2022), constructed a graph neural network based on positional encoding to extract embeddings from drug molecular graphs and miRNA-drug heterogeneous networks. Then, these embeddings of different layers were combined with a layer attention mechanism to learn powerful feature representations. Finally, the potential miRNA-drug resistance association could be discovered *via* a multi-channel neural network consisting of tensor network, tensor decomposition, and the multi-layer perceptron.

Besides, there are some other deep learning models based on varied neural networks algorithm. In Deepthi and Jereesh (2021), firstly, the principal component analysis was applied to reduce the dimensions of features extracted from the integrated similarity pairs of drugs and miRNAs. Then, they trained a convolutional neural network to obtain deep retrieved features and adopted the support vector machine classifier to predict latent association. Meanwhile, based on Long Short-Term Memory (LSTM) (Abdelbaky et al., 2021), proposed an encoder-decoder model that could perform on the character level of a sequence. They utilized the LSTM Sequence Auto-Encoders to obtain feature embeddings of miRNAs and small molecules, and sequence-to-sequence learning with an RNN to encode sequences. The input sequence reproduced by the decoder was based on the outcome of the encoder.

## 5 Conclusion

As the miRNA-related data is explosively growing, developing advanced computational methods for miRNA therapy is not only an opportunity but also a challenge for medical research. Taking advantage of the traditional machine learning method and deep learning method, the discovery of unknown associations among drugs, diseases, and miRNAs could be greatly anticipated. Furthermore, the prediction results of machine learning models could be compared to miRNA-disease/drug associations validated in experimental methods. In this review, we collected commonly used data sources of miRNA-disease and miRNA-drug, which laid a solid foundation for designing feasible prediction models. Various machine learning-based methods were classified into two parts: predicting potential miRNA-disease association and discovering latent miRNA-drug associations, which facilitated exploring miRNA therapy.

Although machine-learning methods have exhibited tremendous potential, it is still a big challenge to accelerate development in miRNA therapy by adopting data-driven computational approaches. This could be improved by utilizing high-quality data resources and integrating domain knowledge when selecting feature to build and verify models. Nevertheless, considering the experimental data might be unavailable for some miRNA, or only a few data points are accessible, reliable models are difficult to construct. Therefore, machine learning approaches like active learning might be a promising strategy to cope with the limitation of available data used to construct reliable prediction models. Meanwhile, generalizability is essential for the widespread application of machine learning approaches, and it could be examined *via* external validation or cross-validation in their proposed model based on machine learning. Recent work adopted anchor regression once a linear shift made training set and test set distributions varied (Rothenhäusler et al., 2018). Different from the “black box” design in which a specific output conducted by a model cannot be explained, machine learning/deep learning models with understandable results or analytical processes are explainable artificial intelligence (Sample, 2017). It is of great importance for domains like miRNA therapy, in which an understandable relationship between outcomes and features is essential. In general, machine learning explainable tools can be mainly divided into two methods: 1) The local model explainability method is helpful to discover which specific features affected a specific decision; 2) The global model explainability method is centered on the features that most affect all decisions or the model’s results. Recently, an emerging field as machine learning fairness has been proposed to study the role of data biases and model biases like race, gender, disabilities and so on, played in the prediction performance in miRNA therapy.
